# Invasive Candidiasis in Infants and Children: Recent Advances in Epidemiology, Diagnosis, and Treatment

**DOI:** 10.3390/jof5010011

**Published:** 2019-01-24

**Authors:** Thomas J. Walsh, Aspasia Katragkou, Tempe Chen, Christine M. Salvatore, Emmanuel Roilides

**Affiliations:** 1 Departments of Medicine, Pediatrics, and Microbiology & immunology, Weill Cornell Medicine of Cornell University and New York Presbyterian Hospital, New York, NY 10065, USA; 2 Department of Pediatrics, Division of Pediatric Infectious Diseases, Nationwide Children’s Hospital and The Ohio State University School of Medicine, Columbus, OH 43205, USA; Aspasia.Katragkou@nationwidechildrens.org; 3 Pediatric Infectious Diseases, Department of Pediatrics, Miller Children’s Hospital and University of California Irvine, Long Beach, CA 90806, USA; TChen@memorialcare.org; 4 Department of Pediatrics, Division of Pediatric Infectious Diseases, Weill Cornell Medicine of Cornell University and New York Presbyterian Hospital, New York, NY 10065, USA; chs2032@med.cornell.edu; 5 Infectious Diseases Section, 3rd Department of Pediatrics, Faculty of Medicine, Aristotle University School of Health Sciences and Hippokration General Hospital, 54642 Thessaloniki, Greece; roilides@med.auth.gr

**Keywords:** candidemia, *Candida* meningoencephalitis, (1→3)-β-d-glucan, T2Candida, PCR, liposomal amphotericin B, micafungin, anidulafungin

## Abstract

This paper reviews recent advances in three selected areas of pediatric invasive candidiasis: epidemiology, diagnosis, and treatment. Although the epidemiological trends of pediatric invasive candidiasis illustrate a declining incidence, this infection still carries a heavy burden of mortality and morbidity that warrants a high index of clinical suspicion, the need for rapid diagnostic systems, and the early initiation of antifungal therapy. The development of non-culture-based technologies, such as the T2Candida system and (1→3)-β-d-glucan detection assay, offers the potential for early laboratory detection of candidemia and CNS candidiasis, respectively. Among the complications of disseminated candidiasis in infants and children, hematogenous disseminated *Candida* meningoencephalitis (HCME) is an important cause of neurological morbidity. Detection of (1→3)-β-d-glucan in cerebrospinal fluid serves as an early diagnostic indicator and an important biomarker of therapeutic response. The recently reported pharmacokinetic data of liposomal amphotericin B in children demonstrate dose–exposure relationships similar to those in adults. The recently completed randomized clinical trial of micafungin versus deoxycholate amphotericin B in the treatment of neonatal candidemia provides further safety data for an echinocandin in this clinical setting.

## 1. Introduction

This paper reviews the recent advances in three selected areas of pediatric invasive candidiasis: epidemiology, diagnosis, and treatment, as presented in a lecture at the 20th Meeting of the International Immunocompromised Host Society. The paper reviews the nationwide secular trends of pediatric invasive candidiasis in the United States and Europe. Our review then further discusses new approaches to laboratory diagnosis and therapeutic monitoring while underscoring the continued need for bedside clinical evaluation. We then further review recent studies in pediatric antifungal pharmacology and therapeutics that provide new insights into safety, tolerability, pharmacokinetics, and efficacy for the management of invasive candidiasis.

## 2. Epidemiology

### 2.1. Secular Trends of Candidemia

Candidemia is the leading cause of invasive fungal infections in hospitalized children. Among the different populations of pediatric patients, the highest rates of candidemia have been recorded in neonates and infants <1 year of age [1,2,3,4]. However, candidemia in pediatric patients is associated with better therapeutic outcomes than in adults. For neonates and young infants, this improved outcome is associated with higher inpatient costs, in comparison with the costs associated with the treatment of adults. Additional comparative data pertaining to pediatric and adult secular trends are depicted at https://www.cdc.gov/fungal/diseases/candidiasis/invasive/statistics.html [5].

During the last decade, there has been a declining secular trend in the incidence of pediatric candidemia the United States and European Union [1,2,3,4,5]. The United States Centers for Diseases Control (CDC) initiated a population-based surveillance of four US metropolitan areas between 2009 and 2015 [5]. The overall incidence of candidemia in neonates decreased from 31.5 cases/100,000 births in 2009 to 10.7 and to 11.8 cases/100,000 births between 2012 and 2015, while the incidence in infants decreased from 52.1 cases/100,000 births in 2009 to 15.7 and to 17.5 between 2012 and 2015. The incidence of candidemia in non-infant children decreased similarly from 1.8 cases/100,000 births in 2009 to 0.8 cases/100,000 births in 2014.

Consistent with these data, there was a decline in the incidence of candidemia in patients who were <1 year in a population-based observational study conducted in Atlanta, Georgia, from approximately 60 per 100,000 person-years in 2008–2009, to less than 40 per 100,000 person-years in 2012–2013. Similarly, there was a decline of approximately 40 per 100,000 person-years in 2008–2009 to less than 20 per 100,000 person-years in 2012–2013. The secular trends in adults were relatively stable.

This decrease in the incidence of pediatric candidemia may be related to several factors regarding the care of central venous catheters [1,2]. These include hospital-wide implementation bundles, guiding insertion and the maintenance of central lines. These measures underscore the importance of using fully sterile barrier precautions, using chlorhexidine in the preparation of the skin during insertion of central lines, taking meticulous care of the catheter and its insertion site, and having daily discussions over the need for a central venous catheter. 

### 2.2. Risk Factors

The risk factors for invasive candidiasis in neonates, particularly in prematurely born infants, warrant special consideration. In a study involving a prospective observational cohort of 1515 extremely low-birth-weight (ELBW) infants, which took place over three years at 19 centers of the US Eunice Kennedy Shriver National Institute of Child Health and Human Development (NICHD) Neonatal Research Network, Benjamin et al. quantified the risk factors predicting infection in high-risk premature infants [3]. Among the 1515 infants enrolled, 137 (9.0%) developed invasive candidiasis, documented by positive culture from one or more of the following sources: blood (*n* = 96); urine obtained by catheterization or suprapubic aspiration (*n* = 52); CSF (*n* = 9); other sterile body fluids (*n* = 10). 

Among the different predictive models that have been developed for invasive candidiasis in neonates, a multivariable analysis of potentially modifiable risk factors associated with candidiasis identified the presence of an endotracheal tube, the presence of a central venous catheter, and a receipt of an intravenous lipid emulsion [3]. A second model predicted candidiasis at the time of blood cultures. Components of the history, physical exam, and initial laboratory evaluation that predicted candidiasis included vaginal delivery, week of gestational age, presence of *Candida*-like dermatitis observed during the physical exam, central venous catheter, lack of enteral feeding, hyperglycemia, number of days of antibiotic exposure in the week prior to culture, and thrombocytopenia [3]. The clinical prediction model had an area under the receiver operating characteristic curve of 0.79 and was superior to clinician judgment (0.70) in predicting neonatal invasive candidiasis.

In this groundbreaking study, invasive candidiasis was found to increase the risk of death in neonates; for example, 47 of 137 (34%) infants with candidiasis died, in comparison with 197 of 1378 (14%) patients without candidiasis (*p* < 0.0001) [3]. Mortality was the highest in the infants from whom *Candida* was isolated from multiple sources. For infant patients with positive urine and blood or positive urine and CSF, the rate of mortality was 16 of 28 (57%). Underscoring the significance of the recovery of *Candida* spp. from urine in neonates, mortality rate was similar in patients who had *Candida* spp. isolated only from blood and those with *Candida* isolated only from urine. 

## 3. Diagnosis

### 3.1. Clinical Diagnosis

The bedside assessment of disseminated candidiasis begins with an understanding of the relative risks and a recognition of its clinical manifestations [6,7,8,9,10]. The clinical manifestations of invasive candidiasis include endophthalmitis (chorioretinal and vitreal lesions), hematogenous *Candida* meningoencephalitis (HCME) (seizures, intraventricular hemorrhages, developmental regression or delays, and CSF pleocytosis), endocarditis (murmurs, peripheral embolic manifestations, and congestive heart failure), hepatosplenic candidiasis (chronic disseminated candidiasis, persistent fever, left upper quadrant or right upper quadrant abdominal pain, and anorexia), acute disseminated candidiasis (multiple cutaneous lesions, diffuse myalgias, hypotension, and multiorgan failure), renal candidiasis (decreasing creatinine clearance, obstructive nephropathy, and renal bezoars), and osteoarticular infections (osteoarticular lesions that are unresponsive to empirical antibacterial therapy). 

### 3.2. Laboratory Detection

Blood culture systems are relatively insensitive in the detection of deeply invasive candidiasis [11]. Non-culture-based methods, such as nucleic acid amplification systems, enzyme immunoassays for circulating mannans, and enzymatic systems for detection of (1→3)-β-d-glucan, are emerging as important laboratory tools for the diagnosis of invasive candidiasis [12,13,14,15,16]. 

### 3.3. T2Candida for Detection of Candidemia

The T2Candida system was recently licensed by the US FDA and was designated as superior to conventional blood culture systems for the detection of candidemia. In detecting the five most commonly recovered medically important *Candida* spp. (*Candida albicans*, *Candida tropicalis*, *Candida parapsilosis*, *Candida glabrata*, and *Candida krusei*), the T2Candida system utilizes a T2 magnetic resonance technology coupled with pathogen-derived nucleic acid amplification to identify the five target pathogens within 2 to 5 h from the time of initiation of the assay. Studies in adults have demonstrated a more rapid time to detection, in comparison with that of conventional blood cultures [15,16]. 

Little is known about the diagnostic utility of the T2Candida system in pediatric patients. In a study conducted at Children’s Hospital of Philadelphia, whole blood from 15 children with candidemia was collected immediately following a blood culture draw [14]. Given the need for conserving the blood volume in this pediatric study, the amount of blood required by the system was reduced by pipetting whole blood directly onto the T2Candida cartridge. The specimens were subsequently run on the T2Dx Instrument (T2 Biosystems). The T2Candida biosystem correctly identified 15 positive and nine negative results within 3 to 5 h. The authors concluded that the T2Candida system was able to efficiently diagnose candidemia in pediatric patients while using low-volume blood specimens. 

### 3.4. CSF (1→3)-β-d-Glucan as a Biomarker for Detection and Therapeutic Monitoring of Candida Infections of the Central Nervous System

HCME in pediatric patients is a life-threatening infection that is fraught with the potential of serious neurologic morbidity if not recognized and treated early [6,17]. HCME is observed in neonates, as well as in children with B-cell acute lymphoblastic leukemia, acute myelogenous leukemia, and primary immunodeficiencies. Associated with seizures, intraventricular hemorrhage, cortical blindness, and neurocognitive impairment, as well as the loss of developmental milestones, the early diagnosis of HCME is difficult, and its recurrence following the completion of antifungal therapy is common. 

Petraitiene et al. originally demonstrated that CSF (1→3)-β-d-glucan levels correlated with CNS tissue infection in experimental HCME [18]. The expression of CSF and plasma (1→3)-β-d-glucan in the non-neutropenic rabbit model of experimental HCME treated with micafungin and with amphotericin B was predictive of the clinical features of this infection. Consistent with clinical observations regarding the difficulty in establishing a microbiological diagnosis, despite a well-established infection throughout CNS tissues, only 8% of CSF cultures were positive in untreated control animals. By comparison, all 25 CSF samples from these animals were found to be positive for (1→3)-β-d-glucan (755 to 7,750 pg/mL) (*p* < 0.001). 

Changes in CSF (1→3)-β-d-glucan levels were highly predictive of antifungal therapeutic response, while clearance of *C. albicans* from blood cultures was not predictive of the eradication of organisms from the CNS [17]. The levels of (1→3)-β-d-glucan in CSF significantly decreased in comparison to those in untreated control animals. The levels of CSF (1→3)-β-d-glucan correlated with therapeutic responses to micafungin in a dose-dependent pattern, with a residual fungal burden in the cerebral tissue (*r* = 0.842). Thus, CSF (1→3)-β-d-glucan levels were predictive biomarkers for the detection and the therapeutic monitoring of experimental HCME. Building upon these data, a clinical trial was designed in the attempt to improve the management of HCME in pediatric patients. 

Salvatore, Chen, and colleagues measured (1→3)-β-d-glucan levels in serially collected samples of serum and CSF of pediatric patients (aged 0–18 years) with a diagnosis of probable or proven HCME and CNS aspergillosis [19]. Among the nine cases of fungal infections of the central nervous system, seven were caused by HCME. All patients at baseline had detectable (1→3)-β-d-glucan in their CSF. In the six patients who completed the therapy for HCME, the elevated CSF (1→3)-β-d-glucan levels decreased to <31 pg/mL. One patient, who was unable to complete the antifungal therapy, died as the result of an overwhelmingly disseminated candidiasis. Monitoring serial CSF (1→3)-β-d-glucan levels in HCME was critical in determining the length of therapy, which ranged from 3 to 6 months, on the basis of individualized assessments. Subsequent reports have confirmed the utility of measuring CSF (1→3)-β-d-glucan levels for initial diagnosis and therapeutic monitoring of HCME [20,21,22]. 

## 4. Treatment

### 4.1. Liposomal Amphotericin B in Immunocompromised Children

Liposomal Amphotericin B (L-AMB) is widely used in the treatment of invasive fungal infections in immunocompromised children; however, little is known about its safety and pharmacokinetics in this vulnerable patient population. Seibel and colleagues therefore conducted a study of the safety, tolerability, and pharmacokinetics of L-AMB in 40 immunocompromised children and adolescents in a sequential-dose-escalation, multidose clinical trial [23]. Ten to 13 patients between the ages of 1 and 17 years were enrolled into each of the four dosage cohorts: 2.5, 5.0, 7.5, or 10 mg/kg, to receive empirical antifungal therapy for persistent fever and neutropenia or for the treatment of documented invasive fungal infections. 

Serum creatinine increased from a mean of 0.45 ± 0.04 mg/dL to 0.63 ± 0.06 mg/dL across all dosage groups (*p* = 0.003). There was a significant increase in serum creatinine in dosage cohorts of 5.0 and 10 mg/kg/day. A greater frequency of hypokalemia and vomiting was also observed in patients receiving 10 mg/kg. Among the 565 infusions, 63 (11%) infusion-related adverse effects occurred. Five patients experienced acute infusion-related reactions at both the 7.5 and the 10 mg/kg dosage levels. 

L-AMB in this patient population exhibited nonlinear pharmacokinetics [24]. The area under the concentration–time curve from 0 to 24 h (AUC_0-24_) on day 1 increased from 54.7 ± 32.9 to 430 ± 566 μg·h/mL in patients receiving 2.5 and 10.0 mg/kg/day. The pharmacokinetic data were best described by a 2-compartment model that incorporated weight and an exponential decay function describing volume of distribution. The population-based model also demonstrated a significant (*p* = 0.004) relationship between the mean AUC_0-24_ and the probability of nephrotoxicity, with an odds ratio of 2.37 (95% confidence interval, 1.84 to 3.22). 

In summary, these data collectively support the use of a range of dosages comparable to those used in adult patients for the treatment of invasive fungal infections, with the understanding that azotemia may occur in direct relation to the AUC_0-24._

### 4.2. Micafungin in Neonates

Extensive preclinical studies in the treatment of experimental disseminated candidiasis [25,26,27,28,29] and clinical studies in pediatric patients [30,31,32,33,34,35,36,37,38,39] supported the investigation of micafungin in neonates in comparison with that of amphotericin B deoxycholate. Benjamin and colleagues compared the efficacy, safety and pharmacokinetics of intravenous micafungin at 10 mg/kg/d with intravenous amphotericin B deoxycholate at 1 mg/kg/d, in a phase 3, randomized, double-blind, multicenter, parallel-group, noninferiority trial, performed on infants >2–120 days of age with proven invasive candidiasis [40]. A total of 20 infants received micafungin, and 10 received amphotericin B deoxycholate. Although the study was terminated early because of low recruitment, fungal-free survival was observed in 12 out of the 20 [60%; 95% CI: 36–81%] infants treated with micafungin, versus 7 of the 10 (70%; 95% CI: 35–93%) infants treated with amphotericin B deoxycholate. The pharmacokinetic-model-derived mean area under the concentration-time curve (AUC) at steady state for micafungin was 399.3 ± 163.9 µg·h/mL, with an AUC pharmacodynamic target exposure of micafungin of 170 µg·h/mL. A population-based pharmacodynamic analysis supported a direct relationship between plasma exposure and the successful eradication of candidemia [41].

### 4.3. Anidulafungin in Pediatric Patients

Building upon earlier preclinical and clinical studies of anidulafungin [42,43], a recently published study reports on the safety and efficacy of anidulafungin in pediatric invasive candidiasis [44]. Anidulafungin was administered at a 3 mg/kg loading dose on day 1 and at a 1.5 mg/kg/d maintenance dose thereafter to patients between the ages of 2 and <18 years. Among 49 patients who received ≥1 dose of anidulafungin for a median 11 days (range of 1–35 days), all were reported to have a treatment-emergent adverse event (AE), such as, most commonly, diarrhea, vomiting, and fever. Treatment was discontinued due to AEs in four cases which were thought to be related to anidulafungin. Among the 48 patients with an isolate of a *Candida* spp., organisms were identified as *C. albicans* in 37.5%, *C. parapsilosis* in 25.0%, *C. tropicalis* in 14.6%, and *Candida lusitaniae* in 10.4%. One patient did not have an isolate of *Candida* sp. recovered. The global response success rate was 70.8%, while all-cause mortality was 8.2% at the end of intravenous therapy and 14.3% at 6-week follow-up. None of the deaths were considered to be treatment-related. The results of this study support the role of anidulafungin at the studied dosages for the treatment of pediatric invasive candidiasis.

## 5. Conclusions and Future Directions

During the past several years, important advances have been achieved in key areas of the epidemiology, laboratory diagnosis, and treatment of pediatric invasive candidiasis. There has been a clear downward trend in the incidence of pediatric invasive candidiasis. Nonetheless, pediatric invasive candidiasis remains an important cause of healthcare-associated sepsis and infectious morbidity. The bedside recognition of invasive candidiasis is challenging, and clinical manifestations are usually non-specific. Advances in the laboratory diagnosis of candidemia and deeply invasive candidiasis are helping to improve the recognition of these serious infections. Among these advances is the T2Candida system, which has the ability to detect more cases of candidemia than conventional blood cultures within 3 to 5 h. Another important advance is the detection of CSF (1→3)-β-d-glucan levels, which are highly sensitive in the diagnosis of HCME and can be serially monitored to guide the duration of and evaluate the response to the antifungal therapy. 

Recently reported studies of L-AMB, micafungin, and anidulafungin in pediatric patients have been have been important advances in the management of invasive candidiasis in infants can children. A major body of preclinical and clinical studies has established the safety, pharmacokinetic, and efficacy profile of these potent antifungal agents in pediatric patients, including those patients with candidemia and other forms of deeply invasive candidiasis. As resistance to echinocandins and antifungal triazoles develops in *Candida* spp., new antifungal agents will be necessary to treat these emerging medically important pathogens. Among the new antifungal agents in development are the first-in-class molecules SCY-078, which is a triterpene inhibitor of (1→3)-β-d-glucan synthase, and APX-001, which is an inhibitor of fungal glycosyl-phosphatidyl-inositol (GPI) biosynthesis [21]. As these agents are developed, well-defined pediatric studies will need to be designed and implemented for the management of pediatric invasive candidiasis and other mycoses.
